# Linking Heat Stress to Impaired Cardiac Repair: The ER Stress–Angiogenesis Axis as a Critical Barrier

**DOI:** 10.3390/ijms27073186

**Published:** 2026-03-31

**Authors:** Tao Cheng, Lu Gan, Rong Yao

**Affiliations:** Department of Emergency Medicine, Institute of Disaster Medicine and Institute of Emergency Medicine, West China Hospital, Sichuan University, Chengdu 640041, China; chengtao@wchscu.edu.cn

**Keywords:** environmental cardiology, heat stress, microvascular rarefaction, ATF6, endothelial memory, fibrosis–conduction mismatch, mitochondria-associated membranes (MAMs)

## Abstract

Climate change has transformed extreme heat from a transient environmental perturbation into a persistent threat that worsens cardiovascular outcomes. Epidemiological studies show a lag between heat exposure and peaks in acute myocardial infarction (AMI) mortality, indicating a subclinical, latent vulnerability. This latent vulnerability likely originates at the level of the microvasculature, as cardiac microvascular endothelial cells (CMECs)—the heart’s primary “thermal sensors”—are uniquely susceptible to proteotoxic stress. The existing literature suggests that this sensitivity may be mediated by thermodynamically gated activation of the activating transcription factor 6 (ATF6) branch of the unfolded protein response (UPR), which could function as a master switch that reprograms endothelial cells from a pro-repair to a maladaptive, anti-angiogenic phenotype. However, this mechanism is derived primarily from preclinical studies and lacks direct validation in humans. The resulting “endothelial memory” is sustained by epigenetic modifications and organelle uncoupling; it persists beyond the initial insult and impairs subsequent neovascularization. As a result, ischemia occurs later in a compromised microenvironment, promoting a fibrosis–conduction mismatch that drives infarct expansion and arrhythmic risk. Thus, the post-exposure latent phase emerges as a novel therapeutic window: Precision targeting of the ER stress–angiogenesis axis during this period offers a focused strategy to protect heat-vulnerable individuals

## 1. Introduction

The interaction of environmental heat stress with cardiovascular disease is one of the most important but often overlooked areas of modern medicine [1,2,3]. The Anthropocene has fundamentally altered the landscape of cardiovascular risk, transforming climate change from a distant ecological abstraction into an immediate physiological threat and a deepening public-health crisis [4,5]. Among its many effects, heat exposure has outperformed other meteorological hazards as the leading cause of weather-related mortality worldwide [6]. Extreme heat events, such as the 2003 European heatwave and the 2023 global extreme heatwave, have triggered massive cardiovascular excess mortality, highlighting the special vulnerability of the cardiovascular system to heat exposure. Although the respiratory systems and renal systems are also affected, the cardiovascular system bears the greatest burden of heat-related morbidity and mortality [6,7,8].

Traditional paradigms have portrayed heat stress mainly as an acute hemodynamic challenge [9]: The need to redistribute central blood volume to the skin for evaporative cooling requires a substantial increase in cardiac output, often doubling or tripling in intensity, which imposes a significant metabolic demand on the myocardium [10]. The supply–demand mismatch model does not account for the time-dependent patterns of recent high-resolution population studies. In particular, the risk of death after acute myocardial infarction (AMI) traces not only peak ambient temperature, but also a distinct lag effect that can persist for days after a heat wave has gone away [11,12].

This temporal disconnect implies that heat stress is not a temporary hemodynamic trigger that resolves with cooling, but an event that leaves a lasting biological “imprint” on the cardiovascular substrate. The persistent risk suggests that heat stress induces subclinical molecular changes that impair the heart’s capacity to repair and adapt [13,14]. Although good epidemiological evidence links heatwaves to worse post-MI outcomes and heart failure, biological transducers that convert heat stress into adverse cardiac remodelling are unclear [15,16,17].

Current paradigms largely focus on the direct effects of thermal stress on cardiomyocytes (e.g., calcium overload or energetic failure) [18,19] but we argue that this view is incomplete. The coronary microvasculature defines the myocardium’s metabolic boundary conditions and coordinates repair, acting as the heart’s “thermal sensor” [20,21]. Here we synthesize scattered preclinical evidence to suggest a unified mechanistic model in which heat may induce a maladaptive form of “endothelial memory” in response to the endoplasmic reticulum stress signalling, by activating the transcription factor 6 (ATF6) axis, which limits the angiogenic plasticity required for post-infarction repair. This renders the heart latently vulnerable. Defining this ER stress–angiogenesis axis is important for the development of therapies that focus beyond simple environmental avoidance and target the root cause of repair failure in a world increasingly prone to heat waves.

## 2. The Clinical Spectrum: From Heat Penalty to Latent Vulnerability

### 2.1. The “Heat Penalty” and Lagged Mortality

Heat stress differs fundamentally from cold stress. While cold stress causes vasoconstriction and plaque rupture through mechanical shear [22,23], heat stress produces the opposite hemodynamic trio: systemic vasodilation, hypotension, and compensatory tachycardia [8,10,24]. In healthy people, this response is manageable physiologically, but in fixed coronary stenosis or early atherosclerosis supply–demand mismatch can be catastrophic [16].

Epidemiological data show a nonlinear J- or U-shaped correlation between temperature and AMI morbidity; mortality risk is increasing, especially when temperatures exceed local limits (i.e., 95th percentile of daily maximum temperature) [2,25,26]. The concept of a “heat penalty” goes far beyond incidence, all the way up to prognosis: Patients who suffer an AMI during or immediately after heat exposure have a far higher in-hospital death rate, cardiogenic shock and complex arrhythmias than patients who had similar infarctions during normothermic periods [23].

Crucially, this risk profile does not normalize immediately after cooling. The “lag effect” refers to a period of high risk lasting 3–7 days after the insult [11,27,28]. If heat were only a trigger, risk would fall once the hemodynamic burden was reduced. The sustained vulnerability implies a cumulative physiological toll (or “molecular scar”) lasting beyond the stressor [29,30,31]. Current risk models (e.g., GRACE or TIMI scores), which use snapshot parameters at admission [32], do not account for this history-dependent vulnerability [33], and are thus a key blind spot in clinical prognosis [3]. The “lag phase” is a period of subclinical pathology where the heart is stable but biologically fragile.

### 2.2. Vulnerability and Synergistic Toxicity

“Vulnerability” denotes a state in which an individual is more susceptible to subsequent insults because of exposure to environmental and social stressors combined with a diminished adaptive capacity [34]. This susceptibility is not uniformly distributed; rather, it is closely associated with biological characteristics and socio-environmental factors. The burden is distributed equally, showing a deep link between biology and environment [35]. Older adults face a “double threat” [28]: Biologically, they have limited thermoregulatory reserves (difficult thirst perception and sweating) and have preexisting endothelial dysfunction, which narrows their homeostatic range [27]. Socially, they are more likely to live in isolation or to lack access to climate-controlled environments [28,36].

**Figure 1:**
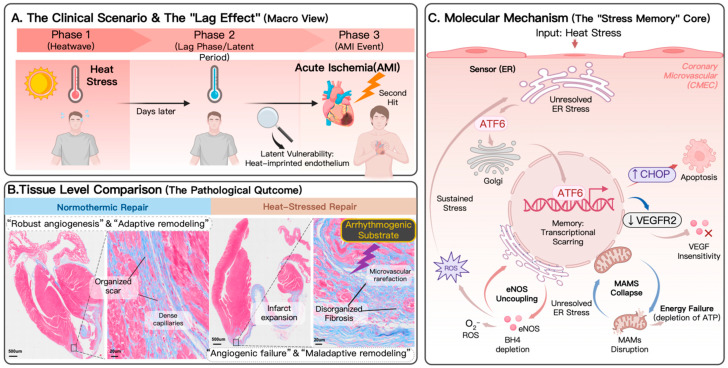
The “heat-imprinted” endothelium: a mechanistic barrier to cardiac repair. (**A**) Clinical temporal dynamics: Heat stress functions as a priming event. Even after ambient temperatures return to normal (the lag phase), the endothelium retains a latent vulnerability that predisposes to worse outcomes following a subsequent AMI. This phase is clinically silent yet biologically active. (**B**) Pathological consequences: comparison of post-MI repair in normothermic and heat-stressed hearts. Heat stress produces pronounced microvascular rarefaction (angiogenic failure) and infarct expansion. A hallmark is the “fibrosis–conduction mismatch”, in which disordered fibrosis forms an arrhythmogenic substrate distinct from the organized scar seen in control hearts. (**C**) Mechanism of “endothelial memory”: In CMECs, heat stress activates the ATF6 branch of the UPR via thermodynamic gating, provoking maladaptive transcriptional reprogramming that represses angiogenic genes (VEGFR2) and induces apoptosis (CHOP). This memory is perpetuated by two reinforcing cycles. First, eNOS-uncoupling generates ROS that sustain ER stress through positive feedback. Second, MAM collapse disrupts ER–mitochondrial communication and impairs bioenergetics. Together, these processes lock the endothelium into a non-reparative state.

Heat rarely occurs in isolation. Heat waves often coincide with stagnation events, promoting the accumulation of fine particulate matter (PM_2.5_) and ozone and producing synergistic toxicity [37,38]. Pollution-induced systemic inflammation appears to lower the threshold for heat-related endothelial injury, thereby increasing the risk of fatal outcomes [39,40]. Consequently, heat stress should not be considered isolated but as a biological multiplier of existing environmental and physiological insults. Clinically, this results in a “primed” patient with reduced physiological reserve, leaving the heart vulnerable to a subsequent ischemic insult (Figure 1A).

## 3. Pathophysiology: Structural and Electrical Consequences

### 3.1. Impaired Repair and Infarct Expansion

The hallmark of heat-associated AMI is catastrophic failure of reparative healing. In experiments, rodents exposed to heat stress before coronary ligation have larger infarcts than normothermic controls, even though they are the same length of ischaemia [2,13,41,42]. This indicates that the “heat penalty” does not stem solely from the magnitude of the initial ischemic insult but from a subsequent failure of the healing response.

Post-MI repair requires tightly orchestrated transition from an inflammatory to a reparative stage. Successful repair forms a compact, collagen-rich scar which preserves ventricular geometry and prevents rupture [43]. Heat stress appears to interrupt repair and promote maladaptive remodelling. Histological analysis shows “loose” disorganized fibrosis with reduced cross-linking and persistent inflammation [44,45]. This structural fragility promotes early dilatation of the ventricular vessels and accelerates heart failure. This is a histological explanation for the higher hospitalization rate reported in clinical studies [42,46].

### 3.2. The Arrhythmic Substrate: Fibrosis–Conduction Mismatch

Beyond structural remodelling, heat stress markedly destabilizes cardiac electrophysiology. Infarcted hearts exposed to heat show prolonged QRS duration and increased QT dispersion [20,47], classical markers of electrical heterogeneity [48]. Although acute ischemia alters ion-channel kinetics (for example, the downregulation of Connexin-43 and the modification of sodium currents) [9,49], the persistence of these arrhythmias points to an underlying structural substrate.

The structural disarray described above creates a different pro-arrhythmic substrate. We propose a “fibrosis–conduction mismatch” in which heat-induced loose fibrosis produces slow-conduction zones that anchor re-entrant circuits [44]. Unlike normothermic infarct, where dense scar is electrically inert and the impulse bypasses cleanly, loose fibrosis of heat-stressed heart forms diffuse heterogeneous mesh in which viable cardiomyocytes are surrounded by collagen [50]. This promotes zigzag conduction, reduces propagation velocity and facilitates micro-re-entry. The anatomical vulnerability lowers the threshold of malignant arrhythmias (ventricular tachycardia/fibrillation), offering a mechanistic explanation for the excess sudden cardiac deaths seen during heatwaves and directly linking pathological remodelling to electrical instability [2,51] (Figure 1B).

## 4. The Cellular Checkpoint: Microvascular Endothelial Dysfunction

### 4.1. Angiogenesis as the Rate-Limiting Step

The failure of repair and the development of loose fibrosis ultimately result from inadequate vascular support and the recovery after injury depends on timely re-establishing of the microvascular network [52,53,54]. After the acute ischemic injury, during the granulation tissue formation phase, endothelial cells proliferate and migrate to revascularize the border zone [55].

This neovascularisation has two important roles. First, it restores oxygen and nutrient delivery to the metabolic penumbra, salvaging hibernating myocardium [56]. Second, and arguably more important, endothelial cells release angiocrine factors (e.g., nitric oxide, NRG-1, VEGF, PDGF) that promote cardiomyocyte survival and modulate fibroblast activity [57,58,59,60]. Therefore, the endothelium should not be viewed merely as a passive conduit for blood but rather as a key regulator of the cardiac repair response.

### 4.2. Thermal Fuse Effect: The Thermal Sensitivity Mechanism of CMECs

Why does this endothelial cell-dominated repair mechanism fail under heat exposure conditions? Recent evidence shows that cardiac microvascular endothelial cells (CMECs) are particularly sensitive to heat stress [20,61], and act as the heart’s “thermal fuse”. In vitro experiments show that CMECs are significantly more susceptible to febrile temperatures (39–42 °C)—mimicking severe heat stroke or high fever—than fibroblasts or cardiomyocytes [21,61].

Heat stress directly inhibits the endothelial cell cycle and breaks the cytoskeleton and inhibits filopodia formation required for migration and tube formation [21]. The downstream consequence is an angiocrine failure [62]: Rather than releasing reparative factors, heat-stressed endothelium adopts a senescence-associated secretory phenotype (SASP) [63,64], characterized by the secretion of pro-inflammatory cytokines (IL-6, IL-1β) and the release of damage-associated molecular patterns (DAMPs) via extracellular vesicles [65,66]. This paracrine shift enhances local inflammation and fails to restrict activation of the fibroblast. At the tissue level, these changes produce severe microvascular rarefaction (decrease in capillary density) in the infarct border area, resulting in a persistent “no reflow” state that sustains chronic ischemia and loose fibrosis phenotype [67].

## 5. Molecular Mechanisms: The “Heat-Imprinted Endothelium”

### 5.1. Thermodynamic Selectivity of ATF6

What biological sensor converts thermal stress into this maladaptive cellular programme? We propose the unfolded protein response (UPR) as the main transducer. The UPR is a conserved signalling network that preserves endoplasmic reticulum (ER) homeostasis during proteotoxic stress. It consists of three canonical branches—PERK, IRE1 and ATF6. Under physiological conditions, these sensors are kept inactive by the chaperone GRP78; on stress, GRP78 dissociates and all three branches are activated to restore protein-folding capacity [68]. Hypoxia and lack of nutrients activate all three as part of a coordinated adaptive response [68], but heat stress in endothelial cells activates the ATF6 branch strongly and selectively [21,69,70,71].

It has been suggested that this specificity may reflect the thermodynamic stability of UPR sensors. Structures show that the luminal domain of ATF6 has an MHC-I-like fold, which is thermally unstable [68,72,73,74]. Based on this, it is plausible that, under heat stress, the luminal domain collapses thermodynamically in a manner distinct from misfolding by chemical stress. The collapse precedes dissociation of the chaperone GRP78 and rapid translocation to the Golgi and subsequent cleavage activation. This proposed “thermodynamic gating” mechanism could make the endothelium hypersensitive to heat and initiates a transcriptional programme different from that produced by ischemia alone [70,75]. Direct experimental validation of this mechanism in the heart is still awaited.

Sustained activation of ATF6 in this setting may override adaptive homeostasis and precipitate a “terminal UPR”. In contrast to the oscillatory switching activation typical of physiological adaptation, heat-locked ATF6 would act as a switch-causing preference for the pro-apoptotic factor CHOP (DDIT3) [75,76]. By provoking pro-apoptotic signals that counterbalance anti-apoptotic Bcl2 family members, this pathway promotes the programmed elimination of stressed CMECs [77]. This mechanism likely accounts for the selective loss of endothelial viability observed during the clinical lag phase (Figure 1C).

### 5.2. The Epigenetic Lock and Organelle Collapse

The persistence of the “lag effect” implies an underlying memory mechanism and the memory may be a function of scarring chromatin [78]. In non-heat-stress models, ATF6 recruits chromatin-modifying factors to target gene promoters [79]. By analogy, heat exposure might provoke epigenetic alterations via the same pathway, reducing endothelial responsiveness to VEGF [80]. However, there is no direct in vivo evidence that heat induces such an “endothelial memory”. If confirmed, this mechanism would explain persistent endothelial unresponsiveness to reparative signals and ongoing dysfunction despite the cessation of heat exposure or the restoration of VEGF levels.

Moreover, the concept of “endothelial memory” may extend beyond the endothelium itself. Pericytes, which closely interact with microvascular endothelium to coordinate vascular stability and reparative angiogenesis, may also acquire similar stress-induced epigenetic alterations following heat exposure [52,81,82,83]. Emerging evidence suggests that pericyte–endothelial crosstalk is critical for post-infarction remodelling, and disruption of this partnership may further impair vascular repair [84,85]. Collectively, these observations point toward a multi-cellular, orchestrated memory phenomenon. From a temporal perspective, short-term memory (days to weeks) may be sustained by post-translational modifications and persistent UPR signalling, whereas long-term memory (months) could involve stable chromatin remodelling that primes the microvascular unit for maladaptive responses upon subsequent challenges [86,87,88].

Simultaneously, the pathology also affects the organelle network, notably the mitochondria-associated membranes (MAMs), which are essential for calcium transfer and cellular bioenergetics. Heat stress promotes the proteasomal degradation of mitofusin-2 (Mfn2), a key attachment to ER and mitochondria [89]. This decoupling has two major consequences. First, impaired Ca^2+^ transfer to the mitochondrial matrix suppresses the TCA cycle and reduces ATP production, undermining the energy required for migration [90]. Second, elevated cytosolic Ca^2+^ activates the NLRP3 inflammasome, thereby sustaining inflammation [91].

Finally, this state is sustained by an eNOS-uncoupling loop. ATF6-mediated suppression of GTP cyclohydrolase 1 (GCH1) depletes tetrahydrobiopterin (BH4), the essential cofactor for endothelial nitric oxide synthase (eNOS)^80^. Without BH4, eNOS becomes uncoupled and switches from producing NO to generating superoxide. The resulting burst of ROS further nitrosylates ER chaperones, impairing the resolution of ER stress [92,93]. This vicious cycle preserves the “stress memory” and thereby blocks vascular repair [94].

## 6. Translational Implications: Targeting the Latent Phase

### 6.1. Redefining Risk Stratification

The concept of “endothelial memory” fundamentally redefines the therapeutic window [95]. Current protocols emphasize cooling during heat stroke or reperfusion during AMI [20,60]. The model [11,12] suggests that the “latent period”—the days following heat exposure but preceding infarction—represents a critical, yet overlooked, opportunity for prophylactic intervention. By analogy with the lasting phenotypic changes induced by shear stress [96], we propose that heat stress also leaves an epigenetic imprint on the endothelium that should be targeted prophylactically.

Risk stratification must incorporate recent exposure history by integrating the exposome into clinical assessment [97]. Patients who present with mild chest pain or with high cardiovascular risk factors during or after a heat wave should be classified as a “high-risk heat-vulnerable phenotype” [3]. In such cases, standard scores (for example GRACE or TIMI) may underestimate the risk of adverse remodelling and arrhythmia because they do not capture subclinical endothelial dysfunction driven by environmental synergisms [37,98].

### 6.2. Precision Therapeutics: Erasing the Molecular Scar

Therapeutically, the ATF6–angiogenesis axis presents tractable targets for “erasing” the molecular scar before the second hit of ischemia occurs:

- **Chemical chaperones:** Agents such as tauroursodeoxycholic acid (TUDCA) and 4-phenylbutyrate (4-PBA) have shown strong efficacy in preclinical models of cardiovascular, metabolic and ischemic stress [93,99]. By stabilizing protein-folding, they may mitigate sustained endoplasmic reticulum stress during the lag phase [100]. However, their clinical utility is limited by poor pharmacokinetics, requiring impractically high millimolar systemic concentrations to reach therapeutic levels in target tissues [72]. Therefore, novel formulations or targeted prodrug strategies will be needed for human use.
- **Precision Inhibition:** The development of selective ATF6 inhibitors, exemplified by Ceapin-A7, is a significant advance [101]. Unlike broad UPR inhibitors, which can be cytotoxic, Ceapin-A7 specifically sequesters ATF6 in the endoplasmic reticulum and prevents its processing [72]. Administered during the high-risk window, such inhibitors could block the ATF6-driven transcriptional reprogramming while preserving the adaptive PERK and IRE1 branches.
- **Nanomedicine:** Looking further ahead, endothelial-targeted delivery systems, such as antibody-conjugated lipid nanoparticles, could be used to deliver siRNA against ATF6 or plasmids encoding Mfn2 specifically to the coronary microvasculature [102]. This strategy has already shown promise in silencing endothelial adhesion molecules to reduce infarct size [103], and therefore could both limit systemic side effects and directly address the root cause of endothelial memory [104].

## 7. Conclusions

Rising global temperatures are creating a growing intersection between extreme heat and cardiovascular disease beyond conventional cardiology. We argue that heat stress is not just an environmental background factor, but a potent biological exacerbation of AMI outcomes. The evidence reviewed here points to the ER stress–angiogenesis axis as a potential critical barrier to heart repair, offering a mechanistic framework for the clinical phenomenon of “latent vulnerability”. According to this model, heat stress may activate a maladaptive ATF6-based transcriptional and epigenetic programme in the microvasculature, leading to converging harms: silencing the angiogenic capacity through chromatin scarring, suppressing the bioenergetics by collapse of mitochondrial–ER contact sites (MAMs) and creating a pro-arrhythmic substrate through fibrosis–conduction mismatch. Together, these processes create conditions that hinder recovery after AMI. Future work should validate these mechanisms in human cohorts using biomarker studies and seek targeted therapies that separate environmental stress from physiological failure. The emerging field of “Climate Cardiology” therefore requires treatment strategies that address both acute cardiac events and the lasting environmental impact on patient biology.

## Data Availability

No new data were created or analyzed in this study.

## Abbreviations

The following abbreviations are used in this manuscript:

AMI	Acute Myocardial Infarction
ATF6	Activating Transcription Factor 6
BH4	Tetrahydrobiopterin
CHOP	C/EBP Homologous Protein (also known as DDIT3)
CMECs	Coronary Microvascular Endothelial Cells
DAMPs	Damage-Associated Molecular Patterns
eNOS	Endothelial Nitric Oxide Synthase
ER	Endoplasmic Reticulum
GCH1	GTP Cyclohydrolase 1
IRE1	Inositol-Requiring Enzyme 1
MAMs	Mitochondria-Associated Membranes
Mfn2	Mitofusin-2
PERK	Protein Kinase RNA-like Endoplasmic Reticulum Kinase
PM2.5	Fine Particulate Matter (<2.5 µm)
ROS	Reactive Oxygen Species
SASP	Senescence-Associated Secretory Phenotype
siRNA	Small Interfering RNA
TUDCA	Tauroursodeoxycholic Acid
UPR	Unfolded Protein Response
VEGF	Vascular Endothelial Growth Factor
VEGFR2	Vascular Endothelial Growth Factor Receptor 2
4-PBA	4-Phenylbutyrate
